# Small but big player: the important role of microRNAs in rice

**DOI:** 10.3389/fpls.2026.1974403

**Published:** 2026-10-05

**Authors:** Shiqi Zhou, Ting Wu, Shilin Wang, Xiaojing Liu, Kerui Huang, Biaolin Hu

**Affiliations:** 1 Rice Research Institute, Jiangxi Academy of Agricultural Sciences, Nanchang, China; 2 College of Life and Environmental Sciences, Hunan University of Arts and Science, Changde, China

**Keywords:** abiotic stress, biotic stress, miRNA, rice breeding, rice development

## Abstract

Rice is a staple food for more than half of the global population. Research on rice crop establishment and tolerance to biotic and abiotic stresses helps enhance rice productivity and safeguard food security. MicroRNAs (miRNAs) regulate diverse biological processes in rice, including growth, development, and stress resilience by modulating different target genes. They coordinate rice growth and development primarily through phytohormone signaling (abscisic acid, auxin, and gibberellin) and metabolic pathways (cell proliferation and fatty acid metabolism). Furthermore, they regulate rice stress tolerance by modulating hormone signaling pathways and metabolic pathways such as reactive oxygen species (ROS) scavenging. Understanding these regulatory networks is crucial for optimizing rice yield and enhancing stress tolerance. This review synthesizes current understanding of the pivotal roles of miRNAs in regulating rice development and adaptive responses to environmental stimuli. It further discusses the emerging prospects of miRNA applications in rice breeding, laying a conceptual foundation for harnessing miRNA pathways to improve rice productivity and stress resilience, thereby contributing to global food security and agricultural sustainability.

## Introduction

Rice (*Oryza sativa* L.), a staple crop for over half of the global population, is crucial for global nutrition (Ishfaq et al., 2023), and the safety of rice production is directly linked to global food security. However, biotic (diseases, pests, etc.) and abiotic (drought, salinity, extreme temperatures, light stress, and heavy metal pollution, etc.) stresses occur throughout the entire growth and developmental cycle of rice (Wu et al., 2024), severely compromising its yield and quality.

Globally, pests and diseases cause a 30.0% (24.6-40.9%) loss in rice yield (Savary et al., 2019). Drought is a major meteorological disaster that poses extreme risks worldwide. As a primary limiting factor for global rice production, it has reduced global yields by approximately 25% over the past two decades (Hu et al., 2025a). Furthermore, rice production requires substantial water resources; in China, 70% of agricultural water is dedicated to rice cultivation (Sun et al., 2022b). Therefore, breeding drought-tolerant rice varieties can simultaneously improve yield stability and water-use efficiency. Soil salinization has become an increasingly severe global issue, with an estimated 20% of irrigated rice cultivation areas affected worldwide (Aycan, 2026). With the increasing extremity of global climate, high-temperature heat stress, low-temperature cold stress, and extreme rainfall occur frequently, heavily impacting rice yield. It is estimated that every 1 °C increase in annual mean temperature would decrease rice yield by 3.2% (Yu et al., 2024). Moreover, studies indicate that rising nighttime temperatures have a more profound impact on rice yield, with each 1 °C increase, grain yield decreases by approximately 10% (Yu et al., 2024). Globally, low temperatures adversely affect approximately 15 million hectares of rice cultivation, limiting its geographical distribution and reducing productivity in high-latitude and high-altitude regions (Pradhan et al., 2019). The economic and agricultural consequences are severe; it has been documented that in major rice-producing countries such as Japan, South Korea, and Brazil, annual yield losses range from 25% to 40% (Kakar et al., 2016). In China alone, low-temperature stress causes annual yield losses of 3 to 5 million tons (Guo et al., 2022). Light serves as a pivotal environmental cue, regulating not only photosynthesis but also overall plant morphogenesis; consequently, light intensity is recognized as a critical abiotic stress factor. During the rainy season in India and Southeast Asia, rice typically suffers a potential yield loss of 40%-50% due to a 40%-60% reduction in light availability (Sekhar et al., 2019). Heavy metal pollution poses a dual threat to rice productivity and grain quality, with cadmium (Cd) and arsenic (As) pollution being particularly severe. A nationwide survey across four major rice-producing regions in China revealed that 33.6% and 6.19% of 113 soil samples (collected from 19 provinces) exceeded regulatory limits for Cd and As, respectively (Mu et al., 2019). Cadmium stress compromises panicle number and seed-setting rate (%), thereby reducing grain yield; it also affects the edible quality of rice and poses severe health risks (Xia et al., 2024). Prolonged exposure to As also severely damages organs such as the skin, stomach, and intestines (Chen et al., 2009), further affecting the economic benefits of food crops. Both biotic and abiotic stresses trigger oxidative stress in rice, posing a major physiological constraint that adversely affects growth, grain yield, and quality (Tavu and Redillas, 2025). and ultimately threatening global food security. For a long time, research on rice stress tolerance has predominantly focused on physiological stress responses, hormone signal transduction, and the cloning and identification of stress-tolerance functional genes, yielding fruitful results (Hou et al., 2025). However, rice stress tolerance is a complex trait synergistically governed by multiple genes; the improvement of a single functional gene is often insufficient to achieve broad-spectrum and highly efficient stress-tolerance effects.

miRNAs are a class of non-coding RNAs approximately 21 nucleotides in length that regulate gene expression at the post-transcriptional level (Hu et al., 2018). miRNAs regulate gene expression in a sequence-specific manner. By binding to fully or nearly fully complementary sequences of target mRNAs, miRNAs direct post-transcriptional gene silencing by triggering target transcript cleavage or translational repression (Campo et al., 2013). Furthermore, as important post-transcriptional negative regulators, miRNAs can simultaneously target multiple genes (Lin et al., 2021). These target genes can regulate not only rice stress tolerance but also its growth and development; thus, individual miRNAs may coordinately regulate multiple agronomic traits. Therefore, miRNA-target interaction modules can be utilized in crop improvement (Tang and Chu, 2017).

This review provides a comprehensive overview of the functional significance of miRNAs in rice developmental processes and adaptive responses to environmental stimuli. It also discusses the emerging prospects of miRNA applications in rice breeding, and by elucidating the complex interactions among miRNAs, gene regulation, and environmental signals, it lays a conceptual foundation for future research utilizing miRNA pathways to enhance crop performance, thereby contributing to global food security and sustainable agriculture.

## The regulatory function of miRNAs in rice development

miRNAs affect various aspects of rice growth and development, such as root development, internode elongation, leaf morphogenesis, floral organ development, fertility, panicle type, tillering, and grain development. By modulating various hormone signaling pathways and sugar synthesis, these miRNA-mediated regulatory modules are integral to rice plant architecture and key agronomic traits, ultimately deciding the plant’s growth adaptability and yield potential.

Root architecture underpins nutrient acquisition and stress adaptation in rice. Studies have shown that the loss of miR444 affects the growth activity, cell division, and elongation of the root apical meristem, thereby causing pronounced developmental defects in both the primary and lateral roots of rice. During the early growth stage, this leads to significant developmental defects in the primary and lateral roots of rice. This is mediated through its targeting of MADS-box transcription factors *OsMADS27* and *OsMADS57*, among which *OsMADS27* is a key regulator of gibberellin (GA) metabolism, nitrate transport, extensin proteins, and auxin signaling-related pathways. Overexpression of *OsMADS27* improves root development and enhances rice tolerance to abiotic stress, whereas its silencing inhibits root growth (Huang et al., 2025). *OsMADS57* participates in the regulation of GA metabolism, brassinosteroid (BR) biosynthesis, and ammonium ion signal transduction. Overexpression of *OsMADS57* results in root elongation and reduced sensitivity to ammonium ions in rice (Huang et al., 2025; Jiao et al., 2020). In addition to miR444, miR390 and miR393 are also involved in rice root development. Under normal growth conditions, miR390 promotes lateral root growth in rice, while miR393 remains in a latent state. However, when rice encounters stress, miR393 is rapidly induced and subsequently suppresses the lateral root-promoting effect of miR390, thereby negatively regulating lateral root growth (Lu et al., 2018). miR156 is crucial in regulating rice growth and development and also modulates rice root development; silencing miR156 leads to a significant reduction in root number in rice seedlings (Zhang et al., 2017a).

Internode elongation is an important agronomic trait in rice that determines culm length, which is closely associated with lodging, panicle exsertion, and responses to flood disasters caused by the frequent occurrence of climate change. In the mutant of the miR172 target gene *sui4* (*shortened uppermost internode 4*), a mutation in the miR172 binding site leads to enhanced *sui4* expression, which reduces cell expansion and cell wall synthesis activity, resulting in shortened internodes (Ji et al., 2019). In addition, miR535 is also involved in regulating rice internode length. The overexpression of miR535 reduces plant height, likely by decreasing the lengths of the first and second internodes through the regulation of its target gene *OsSPL* (*squamosa promote binding protein-like*) (Sun et al., 2019). Mutations in miR396 cause shortened rice internodes, particularly the uppermost internode. This is because miR396 mutations lead to the downregulation of *CYP96B4* (*Cytochrome P450 96B4*) (whose mutation severely represses internode elongation) (Miao et al., 2020).

Leaf morphogenesis, encompassing leaf shape, leaf angle, and growth polarity, governs the photosynthetic efficiency and canopy architecture of rice. with multiple microRNAs—including miR156, miR408, miR393, miR164, miR319, miR160, miR396, miR1848, miR166, miR159, and miR171—participating in this process. The miR156 target gene *OsSPL14* promotes the accumulation of miR408 by binding to the miR408 cis-element, which in turn affects the expression of its target gene *IAA30* (*Indoleacetic acid-induced protein 30*). *IAA30* is involved in auxin signal transduction in rice, and auxin plays an important role in regulating leaf inclination angle. Furthermore, studies have shown that the miR156-*SPL*-miR408-5p-*IAA30* pathway’s regulation of leaf angle is also influenced by the miR393-*TIR1* (*transport inhibitor response 1*)/*AFBs* (*auxin-signaling F-BOX proteins*) module (Rong et al., 2024). miR156 not only participates in leaf angle regulation but is also involved in rice leaf morphogenesis under Rice ragged stunt virus (RRSV) infection. Research indicates that RRSV infection leads to decreased expression of miR164, resulting in enhanced expression of its target gene *OsCUC1* (*cup-shaped cotyledon 1*). *OsCUC1* is a core regulator of rice leaf development, and its function typically depends on dimer formation. Meanwhile, the miR319 target gene *OsTCP1* (t*eosinte branched cycloidea proliferating cell factors 1*) interferes with *OsCUC1* dimer formation, and *OsTCP1* can also interact with miR156 target genes *OsSPL14, 17*, further amplifying the viral effects on leaf morphology (Wang et al., 2025b). Under normal conditions, overexpression of both miR319a and miR319b results in wider leaves (Yang et al., 2013). miR160-*OsARF18* (*Auxin Response Factor 18*), miR1848-*OsCYP51G3*, and miR166-*OsHB4* (*Rice Homeobox 4*) mediate auxin, BR, and cell wall/vascular development-related pathways, respectively, to regulate leaf rolling, leaf angle, and other traits (Huang et al., 2016; Xia et al., 2015; Zhang et al., 2018a); whereas miR396-*OsGRF* (*growth-regulating factor*) regulates leaf elongation by promoting GA biosynthesis (Miao et al., 2020). *STTM159* (short tandem target mimic 159) plants show leaf curling compared to the wild type (Zhang et al., 2017a). *STTM171* plants exhibit a semi-dwarf plant architecture and drooping flag leaves (Zhang et al., 2017a).

The development of floral organs and the processes of pollination and seed set are critical determinants of rice yield and the utilization of heterosis. In rice, *OsMADS1* acts as an upstream regulator of miR172, participating in floral organ development by modulating miR172 and its target gene *AP2* (*apetala 2*) to regulate lemma and palea elongation (Dai et al., 2016). Both the overexpression of miR396 and the knockdown of its target gene, *OsGRF6*, exhibit similar floral organ developmental defects, including open hulls, elongated sterile lemmas, and altered floral organ morphology. This is because the OsmiR396-*OsGRF6* pathway acts synergistically with *OsGIF1* (*growth regulating factor-interacting factor 1*), participating in floret development by targeting *JMJ706* (*jumonji 706*) and *OsCR4* (*crinkly 4*) (Liu et al., 2014). The overexpression of rice-specific miR5506 leads to multiple abnormalities, such as overdevelopment or underdevelopment of the lemma, an abnormal number of floral organs, loss of paniclelet determinacy, and defects in ovule and embryo sac development (Chen et al., 2021b).

Rice fertility is an important trait affecting rice yield and heterosis, and miRNAs are also involved in the regulation of rice fertility. miR159 regulates pollen fertility by cleaving the transcript of its target gene, *SPEAR2* (*sporocyteless ethylene-responsive element binding factor-associated amphiphilic-repression 2*). When *SPEAR2* function is lost, *TCP14*, another target gene of miR319, over-represses the expression of *OsUGP2* (*UDP-glucose pyrophosphorylase 2*), a key gene in starch synthesis, leading to a failure of starch accumulation in pollen, pollen grain shrinkage, and ultimately male sterility (Tao et al., 2025). The loss of miR2118 results in severe male and female sterility, accompanied by obvious morphological and developmental abnormalities in tapetal cells (Araki et al., 2020). The overexpression of miR1848 reduces the expression of its target gene *OsCYP51G3*, resulting in semi-sterile pollen grains (Xia et al., 2015). Transgenic rice lines expressing rice STTM160 exhibit complete male sterility (Zhang et al., 2017a), while STTM1428 plants produce aborted pollen during the pollen maturation stage (Zhang et al., 2017a).

Rice panicle type directly determines the number of grains per panicle and is a core target in yield breeding. miR156 and miR529 regulate the panicle development process in rice by modulating their target *SPL* genes. The expression level of *SPL* genes plays a crucial role in determining plant architecture, as either excessively high or low *SPL* expression leads to a severe reduction in panicle branching. *SPL* genes can regulate the expression of a large number of transcriptional regulators, among which *SPL14* determines panicle architecture through the direct regulation of the miR172/*AP2* and *MADS34*-*RCN1* pathways (Wang et al., 2021b, 2015). *OsMADS17* positively regulates the expression of miR156, and knockout of *OsMADS17* significantly increases the number of grains per panicle and the number of secondary branches (Ming et al., 2023). Inhibition of miR396 activates *OsGRF6* to bind the promoters of *OsTAWAWA1* and *OsMADS34*, thereby promoting panicle development and increasing yield through the auxin pathway (Gao et al., 2016). miR444 positively regulates grain number, thousand-grain weight, and the number of primary branches through auxin and ethylene signaling (Feng et al., 2023). Loss of miR5504 (Wang et al., 2024) and overexpression of miR1863a inhibit panicle development (Hu et al., 2025b); miR398 positively regulates panicle length (Zhang et al., 2017a). The miR168a–*AGO1* (*argonaute 1*) module regulates panicle number and spikelet branching by modulating miR535 (Wang et al., 2021a; Zhang et al., 2024a); miR408 cleaves *OsUCL8* (*Uclacyanin-Like Protein 8*), affecting panicle morphology through copper homeostasis and photosynthetic efficiency (Zhang et al., 2017b); overexpression of miR397 promotes panicle branching (Zhang et al., 2013); and STTM171 plants exhibit a semi-enclosed panicle phenotype (Zhang et al., 2017a).

miRNAs also play important roles in regulating rice tillering. Auxin plays a critical role in regulating rice tillering, and miR156/miR529-*SPL* (Dai et al., 2018; Wang et al., 2021b). miR393 (Li et al., 2016b), miR319-*OsTCP21*/*OsGAMYB* (*Gibberellin myb*) (Wang et al., 2021d), and miR444 (Feng et al., 2023) all regulate tillering through the modulation of auxin signal transduction (**Table 1**). Among these, miR156/miR529 positively regulate tiller number, while miR319 negatively regulates tiller bud height under low-nitrogen conditions and tiller number under high-nitrogen conditions. Furthermore, the *EBT1* gene, composed of miR156B and miR156C, is key to determining the perenniality of wild rice. After flowering, wild rice can “reset” the expression of miR156, allowing it to resume vegetative growth and continuously produce new tillers (Dai et al., 2026). Overexpression of miR168a leads to reduced tiller number and decreased yield, with miR168a balancing tiller development through miR535 (Wang et al., 2021a). STTM166 lines exhibit wider tiller angles (Zhang et al., 2017a). Rice lines overexpressing miR171 are taller and have more tillers compared to the wild type (Zhang et al., 2017a).

**Table 1:** List of miRNAs associated with rice development.

miRNA	Target gene	Pathway	Developmental stage	miRNA–target interaction validation assays	Reference
miR1428			Pollen fertility		Zhang et al., 2017a
miR1432	*OsACOT*	Fatty acid and plant hormone metabolism	Grain size	Transient expression assay	Zhao et al., 2019b
miR156	*OsSPL14*		Root development	RLM-5'RACE	Zhang et al., 2017a; Li et al., 2023
			Panicle branching		Wang et al., 2021b
			Grain size		Li et al., 2023
		Auxin signal transduction	Leaf development		Rong et al., 2024
			Leaf morphology		Wang et al., 2025b
			Leaf morphology		Wang et al., 2025b
	*OsSPL7/2*	Auxin signal transduction	Tillering	Transient expression assay	Dai et al., 2018; Wang et al., 2021b; Dai et al., 2026; Tseng et al., 2023
			panicle branching		Ming et al., 2023
miR529	*OsSPL14*		panicle branching	RLM-5'RACE	Wang et al., 2021b; Li et al., 2023
			Grain size		Li et al., 2023
miR535	*OsSPLs*		Internode elongation		Zhang et al., 2024a; Sun et al., 2019; Zhang et al., 2022
			Grain size		
			panicle branching		Zhang et al., 2024a
			Tillering		Wang et al., 2021a
miR159	*OsGAMYB*		Leaf morphology		Zhang et al., 2017a

	*SPEAR2*	Sodium starch synthesis	Pollen fertility	RLM-5'RACE	Tao et al., 2025
		cell division and mediate auxin and BR signaling	Grain size		Zhao et al., 2023
miR160	*OsARF18*	Starch accumulation	Grain size	RLM-5'RACE	Huang et al., 2016
		Auxin signal transduction	Leaf morphology		
	*Os10g33940*		Pollen fertility		Zhang et al., 2017a
miR164	*OsCUC1*		Leaf morphology		Wang et al., 2025b
			seed vigor		Zhou et al., 2020b
miR166	*OsHB4*	Polysaccharide synthesis	Leaf morphology	RLM-5'RACE	Zhang et al., 2018a
	*Os12g41860*		Tillering		Zhang et al., 2017a
miR167	*OsARF12*	Auxin and BR signal transduction	Grain size	Transient expression assay	Zhao et al., 2023, 2020
miR168a	*AGO1*		Tillering	Degradome-seq	Wang et al., 2021a; Wan et al., 2022
			Grain size		
			seed vigor		Zhou et al., 2020b
miR171			Leaf morphology		Zhang et al., 2017a
			panicle type		
			Tillering		Wang et al., 2021a
miR172	*sui4*	Cell expansion and cell wall synthesis activity	Internode elongation		Ji et al., 2019
			Floral organs development		Dai et al., 2016
		Histone deacetylase	panicle branching		Wang et al., 2015
miR1848	*OsCYP51G3*	Phytosterols and BR biosynthesis	Leaf angle	RLM-5'RACE	Xia et al., 2015
			Pollen fertility		
			Grain size		
miR1863a			Panicle type		Hu et al., 2025b
miR2118			Pollen fertility		Araki et al., 2020
miR319	*OsTCP1*		Leaf morphology		Wang et al., 2025b
	*TCP14*	Sodium starch synthesis	Pollen fertility		Tao et al., 2025
	*OsTCP21*	Auxin signal transduction	Tillering	Transient expression assay	Wang et al., 2021d
	*OsGAmyb*				
	*Os01g0659400Os01g0222001*		Leaf development		Yang et al., 2013
miR390	*OsTAS3*	Auxin binding	Root development	Cleavage activity assay	Lu et al., 2018; Li et al., 2022a
	
miR393	*TIR1*		Leaf development	Degradome-seq	Rong et al., 2024
	*AFBs*				
	*OsAFB2*	Auxin signaling sensitivity	Tillering		Li et al., 2016b
	
miR396	*OsGRF6*	GA response	Floral organs development	RLM-5'RACE	Liu et al., 2014
		Auxin synthesis and signal transduction	paniclelet development		Gao et al., 2016; Zhang et al., 2020a
	*OsGRF4*	GA and BR signaling transduction	Grain size		Li et al., 2025, 2016; Yang et al., 2021; Li et al., 2022a
	*OsGRF8*				
	*OsGRF*	GA biosynthesis	Leaf development		Miao et al., 2020
			Internode elongation		
miR398	*OsCSD2*		panicle length		Zhang et al., 2017a
			Grain size		
miR408	*IAA30*	Auxin signal transduction	Leaf development	5'RACE	Rong et al., 2024
	*OsUCL8*	Copper ion homeostasis	panicle type	Degradome-seq	Zhang et al., 2017b; Wen et al., 2024
			Grain size		Yang et al., 2021
miR444		Ethylene and auxin signal transduction	panicle branching		Feng et al., 2023
			Tillering		
	*OsMADS27*	GA, BRs, and ammonium ion signal transduction	Root development	Degradome-seq	Huang et al., 2025; Jiao et al., 2020; Pachamuthu et al., 2022
	*OsMADS57*	Low sensitivity of ammonium ions			
miR5144	*OsPDIL1;1*	Formation of protein disulfide bonds	Thousand-grain weight	RLM-5'RACE	Xia et al., 2017
miR530	*OsPL3*		Grain size		Sun et al., 2020b
miR5504	*LOC_Os08g16914*	Cell proliferation and elongation	panicle length	RLM-5'RACE	Wang et al., 2024
		Seed protein and amylose accumulation	Grain size		
miR5506	*LOC_Os03g11370*		Floral organs development	RLM-5'RACE	Chen et al., 2021b
miR5519	*RSUS2*	Sucrose synthesis	Grain size	Dual-LUC transient assay	Guo et al., 2024
miR397	*OsLAC*		panicle branching	RLM-5'RACE	Zhang et al., 2013
			Grain size		

miRNAs play crucial regulatory roles in multiple processes of rice grain development and vigor maintenance, thereby affecting yield formation. miR5504, miR160-*OsARF18*, miR408, miR159, miR1432-*acyl-CoA thioesterase* (*OsACOT*), miR397, miR398, and miR535 positively regulate grain size (Wang et al., 2024; Huang et al., 2016; Li et al., 2016a; Zhao et al., 2023, 2019; Zhang et al., 2013, 2017, 2024); miR5519, miR396-*GRF*, miR167-*OsARF12*, miR1848-*OsCYP51G3*, miR530-*OsPL3* (*PLUS3 domain-containing protein*), miR5144-*OsPDIL1;1* (*Protein Disulfide Isomerase-Like 1;1*), miR168a-*AGO1*, miR156/miR529-*OsSPL14*, and miR530-*OsPL3* (*PLUS3 domain-containing protein*) negatively regulate grain size (Guo et al., 2024; Li et al., 2025; Yang et al., 2021; Zhao et al., 2023; Xia et al., 2015; Sun et al., 2020b; Xia et al., 2017; Wang et al., 2021a; Liu et al., 2019; Li et al., 2023; Sun et al., 2019, 2020). Among these, miR5519, miR160-*OsARF18*, and miR5504 regulate grain size by mediating carbohydrate metabolism, whereas miR396-*GRF*, miR159, miR167-*OsARF12*, miR1432-*OsACOT*, and miR1848-*OsCYP51G3* regulate grain size by mediating phytohormone (auxin, BR, GA, etc.) metabolism.

In summary, miRNAs primarily regulate rice growth, development, and yield formation by modulating hormone signaling, particularly auxin signal transduction. For example, the classical miR156/miR529-SPL pathway regulates rice tillering and leaf development by modulating auxin signaling, ultimately affecting rice yield potential. miR160, miR319, miR390, miR393, miR396, and miR444 regulate leaf morphology, grain size, root development, tillering, panicle development, and panicle branching, respectively, via auxin signaling. In addition, miR1848 regulates leaf angle, fertility, and grain shape through BR signaling, while miR396 modulates grain shape, floral organ development, and leaf development via GA and BR signaling (**Table 1**). Notably, miRNAs achieve coordinated regulation of key yield traits—such as plant architecture, panicle architecture, grain shape, and fertility—through interaction networks linking miRNAs with hormone and carbohydrate metabolism. From a breeding application perspective, the precise multi-trait regulation conferred by this regulatory network provides a key theoretical basis for molecular breeding aimed at high yield and superior quality in rice. Targeted editing of these miRNA-target gene modules could enable directional optimization of rice plant architecture, photosynthetic efficiency, grain size, and other traits, thereby increasing yield.

## miRNAs regulate rice responses to biotic stresses

During rice production, yield losses are frequently caused by various biotic stresses (diseases and insect pests). In recent years, an increasing number of reports have demonstrated that miRNAs, as crucial regulators of gene expression, are involved in the immune responses of rice to various pathogens and pests. miRNAs are indispensable regulatory factors in eukaryotic development and defense.

Rice blast is the most serious fungal pathogen in rice production. The miRNAs mentioned earlier, such as miR160 (Li et al., 2014), miR398 (Li et al., 2014), miR159 (Chen et al., 2021a), miR168a (Wang et al., 2021a), miR319 (Zhang et al., 2018b), miR166 (Salvador-Guirao et al., 2018), miR535 (Zhang et al., 2022), miR164 (Wang et al., 2021a), miR156 (Zhang et al., 2020b), miR172 (Wang et al., 2025a), miR167 (Zhao et al., 2020), miR396 (Chandran et al., 2019), miR1432 (Li et al., 2021a), miR444 (Qiu et al., 2025), and miR530 (Li et al., 2021) are involved in both rice growth and development and yield formation, also participate in regulating rice resistance to rice blast. Among them, miR160-*ARF8*-*WRKY45*, miR398, miR166-*EIN2*, miR159, and miR172-*SNB* (*supernumerary bract*) positively regulate rice resistance to rice blast. miR156, miR535, miR168a, miR164, miR319, miR167-*ARF12*, miR396-*OsGRFs*, miR1432, miR444, and miR530 negatively regulate rice resistance to rice blast. Additionally, miR1320, miR162-*OsDCL1* (*dicer-like 1*), miR7695, miR812w, and miR24584 also positively regulate rice resistance to rice blast (Wang et al., 2021a; Li et al., 2020; Campo et al., 2013, 2021; Liu et al., 2025c); miR169-*NF-YA* (*nuclear factor Y, subunit A*), miR11117, miR827, miR1873, miR1871-*OsMFAP1* (*microfibrillar-associated protein 1*), and miR1863a also negatively regulate rice resistance to rice blast (Li et al., 2017; Gao et al., 2023a; Bundó et al., 2024; Campos-Soriano et al., 2020; Zhou et al., 2020a; Li et al., 2022b; Hu et al., 2025b). The target gene of miR160, ARF8 protein, directly binds to the promoter region and inhibits the expression of *WRKY45*, which acts as a positive regulator of rice immunity (Feng et al., 2022). In contrast, miR156 reduces the expression of *SPL14*, affecting the downstream expression of *WRKY45* and negatively regulating rice resistance to rice blast (Zhang et al., 2020b). miR535 also targets *OsSPL4*, which binds to the promoter of the *indole-3-acetic acid-amido synthetase GH3.2* and promotes its expression; this may co-regulate in parallel with the *OsSPL14*-*WRKY45* module (Zhang et al., 2022). miR535 mediates the cleavage of *OsSPL4* mRNA between the 10th and 11th base pairs in the 3’ untranslated region. Overexpression of *OsSPL4* enhances rice blast resistance, accompanied by enhanced immune responses, including increased expression of defense-related genes and H_2_O_2_ accumulation (Zhang et al., 2022). miR168a targets *AGO1*, and weakening the inhibition of *AGO1* expression by miR168a not only enhances rice blast resistance but also promotes increased tillering, shortens the growth period, and increases yield (Wang et al., 2021a). Since *AGO1* is a key element for miRNA binding to target genes, changes in the miR168a-*AGO1* module lead to changes in multiple miRNA-target gene modules. Specifically, the miR168a-*AGO1* module regulates the miR535-*SPL14* module to control rice yield and immunity, the miR164-*NAC11* module to control growth period and immunity, and miR1320 to control immunity. Therefore, manipulating a single miRNA can improve multiple important agronomic traits in rice (Wang et al., 2021a). miRNAs primarily regulate rice blast resistance by modulating metabolic pathways such as plant hormone signal transduction, H_2_O_2_ accumulation, DNA repair, ROS scavenging, and lignin accumulation. miR159a positively regulates rice resistance to rice blast through the suppression of its target genes *OsGAMYB*, *OsGAMYBL*, and *OsZF* (*C3HC4-type domain-containing zinc finger protein*), which regulate GA signaling (Chen et al., 2021a). Transgenic lines overexpressing miR24584-*OsJAZ13* (a transcriptional repressor in the jasmonic acid signaling pathway), miR319-*OsTCP21*, miR444-*HsfA1* (*heat stress transcription factor A-1*), miR167-*ARF12* regulate rice blast resistance through modulating the JA metabolic pathway (Liu et al., 2025c; Zhang et al., 2018b; Qiu et al., 2025; Zhao et al., 2020). Different target genes of miR398 play different roles in resistance. *CSD1* (*copper/zinc superoxide dismutase 1*), *CSD2*, and *SODX* (*superoxide dismutaseX*) regulate H_2_O_2_ accumulation and ROS concentration, negatively regulating disease resistance, but *CCSD* (*copper chaperone for superoxide dismutase*) may enhance rice resistance to rice blast (Li et al., 2019). miR1432 negatively regulates rice resistance to rice blast by inhibiting its target gene *OsEFH1*, which is involved in increasing calcium ion concentration and producing ROS (Li et al., 2021b). miR11117 negatively regulates rice resistance to rice blast by inhibiting its target gene *OsPAO4* (*polyamine oxidase 4*), which is involved in the ROS signaling pathway, polyamine, and ethylene biosynthesis (Gao et al., 2023a). miR7695 enhances rice resistance to rice blast by downregulating its target gene *OsNRAMP6* (*Natural Resistance-Associated Macrophage Protein 6*), which regulates lectin accumulation and iron homeostasis (Campo et al., 2013). miR827 negatively regulates rice resistance to rice blast by regulating phosphate content in rice leaves (Bundó et al., 2024; Campos-Soriano et al., 2020).

Rice sheath blight and false smut are also fungal diseases. miR160 (Feng et al., 2022), miR172 (Wang et al., 2025a), and miR535 (Zhang et al., 2022) have been reported to positively regulate rice resistance to sheath blight, while only miR444 has been found to negatively regulate sheath blight (Feng et al., 2023). In addition, currently only one miRNA has been identified to be involved in regulating resistance to false smut—miR528. It binds to *OsRNS4* (*ribonuclease 4*) and is transported across kingdoms into the infecting *Ustilaginoidea viren*s fungus, silencing *UvSfk1*, a gene essential for the pathogenicity of *U. virens* (Gao et al., 2026).

Bacterial leaf blight, a bacterial disease, is also one of the major diseases threatening rice production. miR160 (Feng et al., 2022), miR172 (Wang et al., 2025a), miR169 (Yu et al., 2018), miR156 (Liu et al., 2019), and miR1432 (Jia et al., 2021) play important roles in regulating rice resistance to bacterial leaf blight. Among them, miR160, miR172, and miR1432 positively regulate rice resistance to bacterial leaf blight by suppressing their target genes, whereas miR169 and miR156 negatively regulate the resistance. miR2118b/n negatively regulates resistance to bacterial leaf streak by negatively regulating multiple *NLR* (*nucleotide-binding domain leucine-rich repeat receptor*) genes (Zhu et al., 2023).

Viral diseases caused by rice stripe virus (RSV), RRSV, and rice grassy stunt virus (RGSV) severely threaten rice production security in China. miR168a negatively regulates rice resistance to RSV by suppressing *AGO1*, whereas *AGO18* protein can protect *AGO1* by competitively binding to miR168a; subsequently, *AGO1* binds to vsiRNAs to silence the viral genomic RNAs, thereby enhancing the antiviral defense response in rice (Wu et al., 2015). miR528 can be competitively bound by *AGO18*, which releases its target gene, *ascorbate oxidase* (*AO*). *AO* regulates redox homeostasis in plants by oxidizing ascorbate, thereby promoting the accumulation of reactive ROS and negatively regulating rice resistance to RSV (Wu et al., 2017). miR444 positively regulates rice resistance to RSV by targeting *OsMADS23*, *OsMADS27a*, and *OsMADS57* (Wang et al., 2016). Rice plants overexpressing miR171b exhibit enhanced resistance to RSV, indicating that miR171b positively regulates rice resistance to RSV (Tong et al., 2017). miR319 negatively regulates rice resistance to RRSV by regulating the expression of its target gene *OsTCP21* (Zhang et al., 2016a), while miR1868.1 positively regulates rice resistance to RRSV (Zhang et al., 2024a). The upregulated expression of miR399d and the suppression of its target gene *OsPHOSPHATE2* (*OsPHO2*) promote Pi accumulation, thereby increasing rice susceptibility to RRSV infection (Lü et al., 2025). miR535 negatively regulates rice resistance to RGSV (Zhang et al., 2024a).

In addition to damage caused by microbes, rice also faces insect pests such as the brown planthopper (BPH). The miR156-SPLs module is also involved in the regulation of rice resistance to BPH; silencing miR156 can enhance rice resistance to BPH by reducing the biosynthesis of jasmonic acid (JA) and jasmonoyl-isoleucine (Ge et al., 2018). miR396 negatively regulates BPH resistance by targeting and regulating *OsGRF8* (Dai et al., 2019). miR159 negatively regulates BPH resistance; its target gene, *OsGAMYBL2*, interacts with the promoter of *OsGS3*, a negative regulator of BPH resistance, and suppresses its expression (Shen et al., 2023). miR162 positively regulates BPH resistance by regulating the expression of its target gene *OsDCL1* (Chen et al., 2023). The overexpression of miR319 negatively regulates BPH resistance by regulating its target gene *OsPCF5* (*proliferating cell factor 5*) (Sun et al., 2024).

## miRNAs regulate rice responses to abiotic stresses

Drought, salinity, extreme temperatures, light stress, and heavy metal pollution pose serious threats to the safe production of rice. Rice has developed a series of mechanisms to respond to various abiotic stresses, among which the miRNA-mediated abiotic stress response network is one of the important mechanisms by which rice responds to various abiotic stresses.

Among all cereal crops, rice is the most sensitive to drought. miR2105-*OsbZIP86* (*basic leucine zipper 86*), miR535, miR1432-*OsCaML2* (*calmodulin-like protein gene*), miR393, miR162-*OsTRE1* (*trehalase 1*), and miR408 negatively regulate drought tolerance in rice by modulating hormone signal transduction (**Table 2**) (Gao et al., 2022; Yue et al., 2020; Luo et al., 2024; Xia et al., 2012; Tian et al., 2015; Sun et al., 2018). The overexpression of miR408 leads to decreased drought tolerance in rice (Sun et al., 2018). Conversely, the co-overexpression of miR397, miR408, and miR528 can improve drought tolerance in rice (Hong et al., 2024). This may be attributed to the fact that 18 out of the 28 *laccase* (*LAC*) family members in rice are potential targets of miR397, miR408, and miR528, and some *LACs* are co-regulated by multiple miRNAs. A broader suppression of these *LACs* genes can more effectively enhance drought tolerance. However, overexpression of miR408 alone leads to upregulation of *LAC5* and *LAC15*, which are targets of miR397, resulting in reduced drought tolerance upon individual miR408 overexpression. Concurrent overexpression of all three miRNAs achieves more comprehensive suppression of *LACs* (Hong et al., 2024). miR166 negatively regulates drought tolerance in rice by regulating its target gene *OsHB4*, which is involved in cell wall formation and vascular development (Zhang et al., 2018a). miR171 positively regulates drought tolerance in rice by regulating the transcription levels of its target genes *SCL6-I* (*scarecrow-like 6-I*) and *SCL6-II*, which participate in flavonoid compound biosynthesis (Um et al., 2022; Zhang et al., 2024b).

**Table 2:** List of miRNAs associated with stress tolerance in rice.

miRNA	Target gene	Pathway	Stress	miRNA–Target Interaction Validation Assays	Reference
miR11117	*OsPAO4*	ROS and ethylene signaling pathway	Fungal pathogens	Transient expression assay	Gao et al., 2023a
miR1320	*OsERF096*	JA-mediated signaling pathway	Cold	RLM-5'RACE, Dual-LUC transient assay	Sun et al., 2022a
			Fungal pathogens		Wang et al., 2021a
miR1432	*OsCaML2*	Cell wall and increase lignin content	Bacterial pathogens	RLM-5'RACE, Dual-LUC transient assay, Mutated target-site complementation	Jia et al., 2021
		MAPK and hormone signaling	Drought		Luo et al., 2024
	*OsACA6*	Inhibit Ca²⁺ ATPase	Cold	5'RACE	Dai et al., 2024
			Salt		
	*OsEFH1*	Ca^2+^ goes up,ROS level	Fungal pathogens		Li et al., 2021b
miR156	*OsSPLs*	GA signaling pathway	Bacterial pathogens		Liu et al., 2019
			Fungal pathogens		Zhang et al., 2020b
			Cold		Cui et al., 2015
		JA biosynthesis	Pest		Ge et al., 2018
		ROS levels	Cd		Lu et al., 2024
	*OsRePRP1.2*	ROS and ABA signaling pathway	Salt	RLM-5'RACE	Shao et al., 2025
miR159	*OsGAMYBL2*		Pest	Dual-LUC transient assay	Shen et al., 2023
		Accumulation of lignin	Fungal pathogens		Chen et al., 2021a
miR160	*ARF8*		Bacterial pathogens		Feng et al., 2022
			Fungal pathogens		
miR162	*OsDCL1*		Fungal pathogens		Li et al., 2020
		Release of 3-hexenal and 3-hexen-1-ol	Pest		Chen et al., 2023
	*OsTRE1*	ABA signal transduction	Drought	RLM-5'RACE	Tian et al., 2015
miR164	*NAC11*		Fungal pathogens		Wang et al., 2021a
miR166	*OsHB4*	Oxidative stress response	Cd	RLM-5'RACE	Ding et al., 2018
		Cell wall formation and vascular development	Drought		Zhang et al., 2018a
	*RDD1*	Photosynthetic activity	Light		Iwamoto, 2022
	*EIN2*	Ethylene steady state	Fungal pathogens		Salvador-Guirao et al., 2018
miR167	*ARF12*	IAA and JA Signaling Pathways	Fungal pathogens	Transient expression assay	Zhao et al., 2020
miR168a	*AGO1*		Fungal pathogens	Degradome-seq	Wang et al., 2021a
			Salt		Wan et al., 2022
			Virus pathogens		Wu et al., 2015
miR169	*NF-YA*	Biomass accumulation, nitrate, and total amino acid content	Bacterial pathogens		Yu et al., 2018
		H_2_O_2_ buildup	Fungal pathogens		Li et al., 2017
	*LOC_Os12g42400*		Heat		Liu et al., 2017
miR171	*SCL6*	Flavonoid compound biosynthesis	Drought		Um et al., 2022
		Chlorophyll accumulation	Virus pathogens		Tong et al., 2017
	*GRAS*	Stomatal development, ABA-dependent pathway	Salt		Yang et al., 2017
miR172	*SNB*	Lignin accumulation	Bacterial pathogens		Wang et al., 2025a
			Fungal pathogens		
	*IDS1*	ROS Steady state	Salt		Cheng et al., 2021
miR1850.1	*NPR3*	ROS clearance, sugar accumulation	Cold	Dual-LUC transient assay	Shen et al., 2024
miR1861e	*OsGST/OsPILS7b*	ROS scavenging and auxin transport	Salt	RLM-5'RACE	Khare et al., 2025
miR1863a			Fungal pathogens		Hu et al., 2025b
miR1868			Virus pathogens		Zhang et al., 2024a
miR1871	*OsMFAP1*		Fungal pathogens		Li et al., 2022b
miR1873	*LOC_Os05g01790*	DNA methylation	Fungal pathogens		Zhou et al., 2020a
miR192			Cd		Ding et al., 2013
miR2105	*OsbZIP86*	ABA Biosynthesis	Drought	RLM-5'RACE	Gao et al., 2022
miR2118	*NLR*		Bacterial pathogens	Protoplast co-transformation	Zhu et al., 2023
miR24584	*OsJAZ13*	JA signaling pathway	Fungal pathogens	Dual-LUC transient assay	Liu et al., 2025b
miR268		H_2_O_2_ and malondialdehyde levels	Cd		Ding et al., 2017
miR319	*OsTCP21*	JA signaling pathway	Fungal pathogens	Transient expression assay	Zhang et al., 2018b
			Virus pathogens		Zhang et al., 2016a
	*OsTCP21/OsPCF6*	ROS removal	Cold		Wang et al., 2014
	*OsPCF5*	phenylpropane synthesis pathway	Pest		Sun et al., 2024
miR390	*OsSRK*		Cd		Ding et al., 2016
miR393	*OsTIR1/OsAFB2*	Auxin signal transduction	Drought	Degradome-seq	Xia et al., 2012
	*OsTIR1*		Salt		Gao et al., 2011
miR396	*GRFs*		Fungal pathogens	RLM-5'RACE	Chandran et al., 2019
		Flavonoid content	Pest		Dai et al., 2019
		H_2_O_2_ accumulation and ROS clearance	Salt		Yuan et al., 2024
		Sugar metabolism pathway	Heat		Yu et al., 2026; Zhang et al., 2020a
miR397	*OsLAC*	Photosynthetic intensity	Light		Lian et al., 2024
miR3979		Oxidase activity	As		Zhang et al., 2016b
miR398	*CSD1-2/CCS*		Salt		Lu et al., 2022
	*CSD1-2/SODX/CCSD*	H_2_O_2_ accumulation, ROS concentration	Fungal pathogens		Li et al., 2014
miR399d	*OsPHO2*	Accumulation of Pi	Virus pathogens		Lü et al., 2025
miR408	*OsIAA6*		Drought		Sun et al., 2018
	*UCL*	Energy balance	Heat	5'RACE	Wen et al., 2024; Huang et al., 2024
			Light		
miR444	*OsMADS23/OsMADS27a/OsMADS57*		Virus pathogens	Degradome-seq	Wang et al., 2016; Pachamuthu et al., 2022
	*HsfA1*	JA synthesis	Fungal pathogens	RNA pull-down	Qiu et al., 2025
miR5144	*OsPDIL1;1*	Formation of protein disulfide bonds	Salt	RLM-5'RACE	Xia et al., 2017
miR528			As		Liu et al., 2015
	*LOC_Os08g42640*	ROS signals	Cd	RLM-5'RACE	Fu et al., 2025
	*AO*	Ascorbic acid metabolism, ROS clearance	Salt		Wang et al., 2021c
		Redox homeostasis	Virus pathogens		Wu et al., 2017
	*OsRNS4*	Extracellular vesicles	Fungal pathogens		Gao et al., 2026
miR535	*OsSPL7*		Cd	RLM-5'RACE	Yue et al., 2023
		ROS level	Cold		Sun et al., 2020a
		ABA tolerance	Drought		Yue et al., 2020
	*OsSPL14*		Fungal pathogens		Zhang et al., 2022
			Salt		Yue et al., 2020
			Virus pathogens		Zhang et al., 2024a
			Fungal pathogens		Li et al., 2021a
miR5810	*OsMRLP6*	Chloroplast development	Light	RLM-5'RACE	Gao et al., 2023b
miR7695	*OsNRAMP6*	Plant lectins, iron homeostasis accumulation	Fungal pathogens	RLM-5'RACE	Campo et al., 2013; Sánchez-Sanuy et al., 2019
miR812w		DNA Methylation	Fungal pathogens		Campo et al., 2021
miR827	*OsSPX-MFS1 * /*OsSPX-MFS2*	Phosphate content	Fungal pathogens		Bundó et al., 2024

High salinity is a major factor limiting rice growth, thereby leading to reduced crop yields. miR168a-*AGO1*, miR396-*GRF6*, miR156-*OsRePRP1.2* (*repetitive proline-rich protein 1.2*), miR1861e-*OsGST* (*glutathione S-transferase*)/*OsPILS7b* (*auxin efflux carrier (PIN) like protein*) negatively regulate salt tolerance in rice (Wan et al., 2022; Yuan et al., 2024; Shao et al., 2025; Khare et al., 2025). miR172-*IDS1* (*indeterminate spikelet 1*), miR1861h, and miR528-*AO* positively regulate rice salt tolerance (Cheng et al., 2021; Ai et al., 2021; Wang et al., 2021c). All the aforementioned miRNAs regulate salt tolerance by modulating ROS scavenging (**Table 2**). Interestingly, although miR1861e and miR1861h belong to the same family, they exert opposite regulatory effects on salt stress. In addition, miR171c negatively regulates salt tolerance at the germination and seedling stages by regulating stomatal development and the ABA-dependent pathway (Yang et al., 2017). The overexpression of miR393 negatively regulates rice tolerance to saline-alkaline stress (Gao et al., 2011). Inhibiting or knocking out miR535 in rice enhances plant tolerance to NaCl (Yue et al., 2020). miR398 negatively regulates rice salt tolerance by suppressing the expression of its target genes (Lu et al., 2022). The overexpression of miR1432 and the suppression of its target gene *OsACA6* (*calcium-transporting ATPase 6*) lead to decreased plant tolerance to salt stress (Dai et al., 2024). Salt tolerance is enhanced by the downregulation of miR5144-3p or the overexpression of *OsPDIL1;1*. Compared with the wild type, these transgenic rice plants exhibit strong resistance to salt and mercury stresses (Xia et al., 2017). miRNAs play a central role in the response of rice to extreme heat or cold. miR1850.1 negatively regulates cold tolerance in rice by targeting and suppressing the immune gene *NPR3* (*NPR1 homolog 3*) through transcript cleavage and translational inhibition (Shen et al., 2025). The overexpression of miR535 inhibits early seedling growth under cold stress (Sun et al., 2020a). In plants overexpressing miR319b, the downregulation of its target genes *OsPCF6* and *OsTCP21* enhances plant tolerance to cold stress (Wang et al., 2014). These miRNAs and target genes regulate cold tolerance in rice by modulating ROS scavenging. miR1320 positively regulates cold tolerance in rice by suppressing its target gene *OsERF096* (*ethylene response factor 96*) (Sun et al., 2022a). The co-overexpression of three copper-related miRNAs (miR397, miR408, and miR528), which simultaneously silenced multiple laccase-related target genes, significantly enhanced cold tolerance in rice (Hong et al., 2024). Knocking out miR1868 enhances cold tolerance in rice (Shen et al., 2024). Under cold stress conditions, the overexpression of miR156 suppresses the early growth of seedlings (Cui et al., 2015). The overexpression of miR1432 and the suppression of *OsACA6* lead to decreased plant tolerance to cold stress (Dai et al., 2024). miR408 improves rice yield under both normal and high-temperature conditions by cleaving *UCL7* (*Uclacyanin 7*). This is because miR408 and its target gene *UCL7* are involved in regulating energy homeostasis to achieve a balance between growth and heat response. This regulation involves modulating H_2_O_2_ levels and optimizing the relationship between the target of *rapamycin* (*TOR*) and *SNF1-related protein kinase 1* (*SnRK1*) in rice (Wen et al., 2024). The miR396-GRF7 module positively regulates heat tolerance at the seedling and flowering stages: the miR396f knockout line is sensitive to heat, whereas the GRF7 knockout line exhibits enhanced heat tolerance (Yu et al., 2026). Overexpressing miR169 to suppress the expression of its target gene *LOC_Os12g42400* can enhance heat tolerance in rice at the flowering stage (Liu et al., 2017). Overexpressing rice miR393a in creeping bentgrass (*Agrostis stolonifera*) plants can improve heat tolerance (Zhao et al., 2019a).

Light stress poses a serious threat to rice production. Overexpression of miR5810 or downregulation of *OsMRLP6* (*malectin-like protein 6*) in rice leads to reduced expression of genes related to chloroplast development and photosynthesis, decreased net photosynthetic rate, and ultimately results in reduced shoot biomass and grain yield (Gao et al., 2023b). Overexpression of miR408, which targets *UCL*, improves photosynthesis (Wen et al., 2024). Suppressing the pairing between miR166 and *RDD1* (*rice dof daily fluctuations 1*) transcripts (i.e., R1-Cas plants) enhances *RDD1* transcript levels, NH_4_^+^ and PO_4_³^-^ uptake, and photosynthetic activity in rice under elevated CO_2_ conditions (Iwamoto, 2022). miR397, by silencing *OsLAC* (*laccase*) expression, causes rice to exhibit higher photosynthetic intensity, lower photorespiration rates, early maturation, and increased yield (Lian et al., 2024).

Cd and arsenic (As) are highly toxic to both plants and humans. The functional small peptide miPEP156, encoded by pri-miR156, regulates the expression of miR156 and its target *SPLs*, influencing miR156-mediated Cd tolerance in rice. Both exogenous application of synthetic miPEP156 and overexpression of miPEP156 reduce Cd uptake and accumulation in rice, decrease reactive ROS levels, and thereby enhance Cd tolerance (Lu et al., 2024). Knockout of miR535 improves Cd tolerance and reduces Cd content in brown rice, primarily through the regulation of *SPL7* (Yue et al., 2023). Overexpression of miR166 in rice enhances Cd tolerance, which is associated with alleviated Cd-induced oxidative stress in transgenic plants. Furthermore, overexpression of miR166 reduces Cd translocation from roots to shoots and Cd accumulation in grains. Silencing its target gene *OsHB4* via RNA interference enhances Cd tolerance in transgenic rice plants (Ding et al., 2018). Transgenic rice plants overexpressing miR390 exhibit reduced Cd tolerance and higher Cd accumulation compared to the wild type (Ding et al., 2016). Overexpression of miR192 delays seed germination and seedling growth under Cd stress (Ding et al., 2013). Overexpression of miR268 inhibits the growth of rice seedlings under Cd stress, with transgenic plants showing increased hydrogen peroxide and malondialdehyde contents in leaves and elevated Cd accumulation in seedlings (Ding et al., 2017). *UCL23* (a small copper protein) plays a critical function in regulating Cd uptake, tolerance, and accumulation through the modulation of ROS signaling in rice. Additionally, *WRKY51* binds to the promoters of *UCL23* and miR528, thereby promoting Cd regulation in a dual regulatory manner. Natural variation in *UCL23* is important for differential Cd accumulation in rice grains; indica rice varieties carrying the predominant japonica haplotype of *UCL23* exhibit significantly reduced root Cd uptake and grain Cd accumulation (Fu et al., 2025). Transgenic plants overexpressing miR528 are more sensitive to arsenic than wild-type rice (Liu et al., 2015). Transgenic plants overexpressing miR3979 are more sensitive to arsenite, with significantly enhanced As uptake and translocation capacity; under As stress, these transgenic plants show significantly increased proline and soluble protein concentrations in roots, but the activities of various antioxidant enzymes are significantly inhibited (Zhang et al., 2016b).

## Emerging prospects of miRNAs in rice breeding applications

Traditional breeding often faces the dilemma of “unable to have one’s cake and eat it too.” However, miRNAs can simultaneously target multiple genes, and these target genes can regulate not only rice stress tolerance but also its growth, development, and yield. As summarized in the aforementioned regulatory modules (miR156-*SPL*, miR396-*GRF*, miR168a-*AGO1*, miR535-*SPL14*, and other core modules), a single miRNA often simultaneously regulates rice growth, yield formation, and biotic/abiotic stress responses. For instance, it has been reported that specific modulation of miR168a and miR530 expression holds promise for achieving comprehensive improvement of multiple agronomic traits, including increased yield, enhanced disease resistance, and early maturity (Wang et al., 2021a; Li et al., 2021a). Suppression of miR167 expression significantly increases rice grain length, grain width, grain thickness, and grain weight, with field yield improved by 12.90%-21.94% (Zhao et al., 2023). Suppression of miR167 expression also enhances rice blast resistance (Zhao et al., 2020); MIM535 plants maintain an unchanged tiller number but produce larger panicles with more grains, resulting in higher yield per plant than the control, and miR535 negatively regulates rice disease resistance (Wang et al., 2021a; Zhang et al., 2024a, 2022). Suppression of miR396 or miR1432 can improve yield and disease resistance, but at the cost of reducing heat tolerance and drought tolerance, respectively (Chandran et al., 2019; Yu et al., 2026; Zhao et al., 2019b; Li et al., 2021b; Luo et al., 2024). Blocking miR156 expression results in transgenic lines exhibiting enhanced blast resistance without yield penalty—although the number of secondary branches per panicle is reduced, this is compensated by enlarged grains (Zhang et al., 2020b). Knockout of miR1863a can achieve disease resistance without yield loss; however, miR1863a expression levels must be maintained within an optimal range to balance resistance and yield, otherwise, yield reduction occurs (Hu et al., 2025b). By fine-tuning the expression of endogenous miRNAs, the bottlenecks that are difficult to reconcile in traditional breeding, such as the trade-offs between high yield and disease resistance, or between perenniality and high yield, can be elegantly broken (Chandra et al., 2026). However, miRNA-mediated trait regulation is not universally beneficial. For example, overexpression of miR159 improves disease resistance but causes sterility and reduces yield (Chen et al., 2021a). Overexpression of miR162 significantly enhances rice resistance to blast, but the seed-setting rate declines, resulting in a slight reduction in yield per plant (Li et al., 2020). This provides a means to weigh yield against stress tolerance during breeding. Effective strategies include spatiotemporal-specific regulation to activate resistance only when needed, and selecting elite haplotypes with appropriate miRNA expression levels caused by natural variation in promoter regions for molecular-assisted breeding, thereby achieving both high yield and stress resistance.

Spatiotemporal-specific regulation requires the use of modern molecular technologies to modulate miRNA expression. Currently, methods for regulating miRNA-target gene pathways are primarily categorized into those that increase the abundance of specific miRNAs to bind to target genes and inhibit their expression, such as artificial miRNAs (amiRNAs) (Thiebaut et al., 2026) and virus-mediated miRNA overexpression (Zheng et al., 2017); and target mimicry techniques (Franco-Zorrilla et al., 2007) and STTM (Guo et al., 2023), which prevent miRNAs from normally inhibiting target gene expression by competing for miRNA binding. MicroRNA-induced gene silencing (MIGS) is an alternative approach for silencing genes based on the ability of some microRNAs (miRNAs)—a class of sRNAs originating from endogenous transcripts with foldback structures—to induce the production of a new wave of sRNAs (secondary sRNAs) (Gauthier et al., 2026). Herein, Zhao et al. (2019b) placed STTM1432 under the control of the Gt13a promoter (an endosperm-specific promoter). The Gt13a promoter drives downstream gene expression exclusively in the developing grain endosperm while remaining inactive in other tissues (He et al., 2011). Therefore, this approach can enhance yield without compromising drought tolerance. Chen et al. (2013) utilized the native miR528 precursor as a backbone to construct amiRNA precursors. In transgenic rice, expression of these amiRNA precursors driven specifically by leaf-specific promoters derived from either rice or *Arabidopsis* enhanced resistance to bacterial blight without affecting important agronomic traits such as fertility of the transgenic rice. However, owing to the controversies surrounding transgenic biosafety, these technologies are currently used mainly for miRNA functional validation in laboratory settings and are rarely applied in breeding programs.

By contrast, gene editing with the CRISPR-Cas9 system allows subsequent removal of the CRISPR/Cas9 transgene through genetic segregation, backcrossing, and the transgene killer CRISPR (TKC) technology (He et al., 2019), thereby gaining greater public acceptance. CRISPR-Cas9 technology allows for targeted modification of miRNA key regions; however, this method typically generates only 1 bp to 3 bp nucleotide insertions or deletions, which may fail to achieve loss-of-function of plant miRNAs. The CRISPR-Cas12a method developed by Zheng et al. (2025) can generate larger deletions, significantly disrupting the secondary structure of pre-miRNAs and blocking the production of mature miRNAs. Researchers tested the efficacy of the CRISPR-Cas9 and CRISPR-Cas12a systems in knocking out the miR390 precursor in rice, and found that although Cas12a exhibited significantly lower regeneration efficiency than Cas9, it could disrupt miRNA function more thoroughly than the Cas9 system (Zheng et al., 2025). This method has been applied to the editing of multiple miRNA precursors and is relatively well-established. Liu et al. (2025b) developed a plant circRNA-specific knockdown tool based on the CRISPR-Cas13d system, which competitively binds to rice miR398b. Thereby alleviating the post-transcriptional repression of genes mediated by miR398b. This molecular interaction significantly reduces the level of ROS in rice upon infection by *Xanthomonas oryzae pv. oryzae*, thereby negatively regulating rice resistance to bacterial blight. This method exhibits high knockdown efficiency. However, since Cas13d could potentially display collateral activity (javascript):;, it is crucial to select a target that is not overly abundant (Liu et al., 2025b). This method has rarely been applied to date. Liao et al. (2026) reported that CRISPR-Cas9-based glycosylase base editors (gBEs) primarily function as efficient polynucleotide deletion editors (gMDEs) in plants, capable of efficiently generating 6–20 bp deletions in the non-coding regions of rice and soybeans. This method exhibits a low off-target rate. By disrupting the miR156 binding site in the 3’UTR of *OsGLW7*, which encodes *OsSPL13*, researchers enhanced *OsGLW7* expression, thereby increasing grain length, thousand-grain weight, and the grain length-to-width ratio, while grain width remained unaffected (Liao et al., 2026). However, few application examples of this method for editing miRNA target sites in rice have been reported. Liu et al. (2025a) developed a conditional gene knockdown platform based on endogenous miRNAs called MiRKD (miRNA-mediated in-locus gene knockdown). Using this technique, the research team artificially inserted the target sequence of miR396 into the 3’UTR of the *GID1* gene. In this way, the *GID1* transcript, which originally did not carry a miR396c target site, was converted into a “disguised target.” Because miR396 exhibits a tissue-specific expression pattern, this artificial design enabled *GID1* to be knocked down specifically in certain tissues while remaining unaffected in others. This method relies entirely on endogenous miRNAs and does not require the introduction of exogenous proteins or RNA; by editing the 3’UTR of the gene itself, the natural structure of the target protein can be completely preserved. Furthermore, it can be combined with target sequences of different miRNAs to achieve simultaneous regulation of multiple genes, providing a safe and precise new tool for the fine improvement of complex traits. This method has been successfully employed to construct three spatiotemporally controllable gene expression systems: constitutively expressed miR156a, shoot-apex tissue—specific miR396c, and long-day—inducible miR528. However, this method relies on endogenous miRNA expression patterns; therefore, systematic and comprehensive preliminary validation of the tissue specificity and spatiotemporal expression profiles and stress-responsive regulation of the target miRNAs is required before editing targets can be precisely designed and directional trait improvement achieved. Since endogenous miRNAs may exhibit inconsistent expression changes across different rice varieties and under varying environmental conditions (Payne et al., 2026), the application of this method is subject to certain limitations in complex field breeding environments.

In addition, utilizing natural variation for molecular marker-assisted breeding can also regulate miRNA expression. This is also one of the most widely accepted breeding approaches. miR168a negatively regulates various phenotypes, including seed vigor, panicle architecture, tillering, salt tolerance, and resistance to RSV and rice blast. The promoter region of rice miR168a contains abundant natural variations, which can be classified into different haplotypes leading to differences in miR168a expression levels (Zhao et al., 2025). Introducing the haplotype that causes downregulation of miR168a expression into various rice varieties through molecular breeding may improve these agronomic traits. The promoter region of miR528 exhibits typical differentiation between rice *indica* and *japonica* subspecies and wild rice; the miR528-A1 allele is predominantly found in wild rice and indica rice, while the miR528-A2 allele is mainly present in *japonica* rice. The miR528 level in rice lines containing the miR528-A1 allele is significantly higher than that in lines containing the miR528-A2 allele (Yang et al., 2019). miR528 positively regulates resistance to rice leaf streak, RSV, drought, cold, and salt, while negatively regulating resistance to Cd and As. Interestingly, miR528 levels are higher in leaves than in roots. This characteristic is maintained across all haplotypes. Since Cd and As are mainly absorbed by the roots, the miR528-A1 allele can enhance plant resistance to drought, cold, salt, and RSV, while exerting little effect on Cd and As uptake. The miR408 promoter exhibits eight haplotypes (three *japonica*-specific and five *indica*-specific) (Balyan et al., 2023). These haplotypes also possess breeding potential. Different haplotypes of the transcription factors MADS51 and ESR1 show differences in their regulatory activity on the miR1863a promoter, resulting in different expression levels of miR1863a (Hu et al., 2025b); miR1863a negatively regulates panicle number and rice blast resistance. Screening, through molecular breeding and other approaches, varieties with optimal miR1863a expression are expected to attain both high yield and disease resistance. *OsMADS17* positively regulates miR156 expression; in tropical japonica rice, a superior haplotype of *OsMADS17* leads to downregulated *OsMADS17* expression, which in turn reduces miR156 expression and increases the number of spikelets per panicle (Ming et al., 2023). Conserved structural elements in precursor miRNAs regulate the abundance of mature miRNAs; a conserved SNP site (rs10234287911, G/A) located in the miR396c precursor sequence affects the abundance of mature miR396c (Jaganathan et al., 2023). miR396c negatively regulates rice grain size, salt tolerance, and rice blast resistance. Rational application of this SNP locus could enable the creation of rice varieties with both high yield and stress resistance. A GG/AA polymorphism in the loop region of the miR2923a precursor is associated with grain length and length-to-width ratio (Wang et al., 2013). miR156b and miR156c together constitute a locus named “*Endless Branches and Tillers 1*” (*EBT1*), which is responsible for inflorescence reversion and vegetative propagation, thereby promoting the perennial growth of wild rice. In cultivated rice, this locus is “locked” by a repressive histone modification (H3K27me3), whereas in wild rice, the chromatin at this locus is in a more “open” state with lower levels of H3K27me3. By pyramiding *EBT1* with two known rice prostrate growth genes, “wild-rice-like” plants that successfully recapitulate the weedy phenotype of wild rice were developed (Dai et al., 2026). The discovery of these elite haplotypes provides valuable natural germplasm resources for rice to break through the bottleneck in traditional breeding, where yield and stress resistance are difficult to improve simultaneously. In contrast to the severe pleiotropic effects and yield penalties caused by amiRNAs, miRNA overexpression, or miRNA silencing, elite haplotypes can substantially enhance stress resistance through mild and spatiotemporally specific regulatory mechanisms, while maximally preserving normal growth, development, and yield potential of rice. They therefore serve as superior genetic materials—efficient, safe, and directly applicable to molecular marker-assisted breeding—for developing elite rice varieties with high yield, strong resistance, and broad adaptability.

## Conclusion

The roles of miRNAs in rice growth, development, and tolerance to biotic and abiotic stresses are crucial for understanding the complex regulatory networks governing these biological processes. As key post-transcriptional regulators, miRNAs modulate gene expression at the post-transcriptional level, thereby affecting rice growth, development, and stress adaptation. A single miRNA often regulates multiple rice traits. This may be attributed to the fact that miRNAs regulate different target genes, thereby affecting distinct metabolic pathways and consequently exerting different impacts on various traits (**Tables 1**, **2**). As a result, they may positively regulate certain traits while negatively regulating others. Alternatively, a miRNA may regulate the same target gene, yet this target gene exerts different effects on different traits. This review summarizes the regulatory networks of known and functionally validated miRNAs involved in important agronomic traits of rice (**Figure 1**). Numerous canonical regulatory modules exist in rice. miRNA-target gene modules, such as miR156-*SPL*, miR535-*SPL*, miR168a-*AGO1*, and miR396-*GRF*, regulate rice growth and development through hormone signaling and mediate resistance to both biotic and abiotic stresses via pathways including JA biosynthesis and ROS scavenging (**Tables 1**, **2**). However, altering miR156-*SPL* expression improves resistance at the expense of yield reduction (Liu et al., 2019), highlighting the need to balance yield and stress resistance. An effective approach is spatiotemporal-specific regulation: by achieving high or low expression of these miRNAs in specific tissues while leaving their expression unchanged in other tissues, it is possible to increase yield without compromising stress resistance. miRNAs mutually regulate common agronomic traits. Various miRNAs form interactive networks that coordinately regulate agronomic traits. Under stress, miR393 represses miR390 and *TIR1* to modulate auxin signaling, root development, and miR156 expression (Lu et al., 2018). *OsSPL14* (miR156 target) activates miR408 to regulate *IAA30* and leaf angle (Rong et al., 2024). Crosstalk between miR156-*OsSPLs* and miR319-*OsTCP1* impairs *OsCUC1* dimerization, enhancing RRSV-triggered leaf abnormalities (Wang et al., 2025b). miR159-*SPEAR2* remodels miR319-*TCP*-*OsUGP2* expression to induce male sterility (Tao et al., 2025). miR156/miR529-*SPL* directs panicle architecture via miR172/*AP2* and MADS34-*RCN1* pathways (Wang et al., 2021b, 2015). The miR168a-*AGO1* module synchronously shapes multiple agronomic traits: it controls yield/immunity through miR535-*SPL14*, growth duration/immunity via miR164-*NAC11*, and immunity through miR1320 (Wang et al., 2021a). The miR168a promoter harbors natural variation, and the introduction of low-expression alleles through molecular breeding may simultaneously improve multiple agronomic traits. Deepening our understanding of miRNA regulatory mechanisms, mining elite miRNA haplotypes, and employing modern breeding technologies to modulate the expression levels of specific miRNAs in rice can achieve synchronous improvement of multiple traits—for example, obtaining both high yield and disease resistance simultaneously. Based on this, it is expected to enhance rice yield and stress resistance, thereby contributing to global food security.

**Figure 1:**
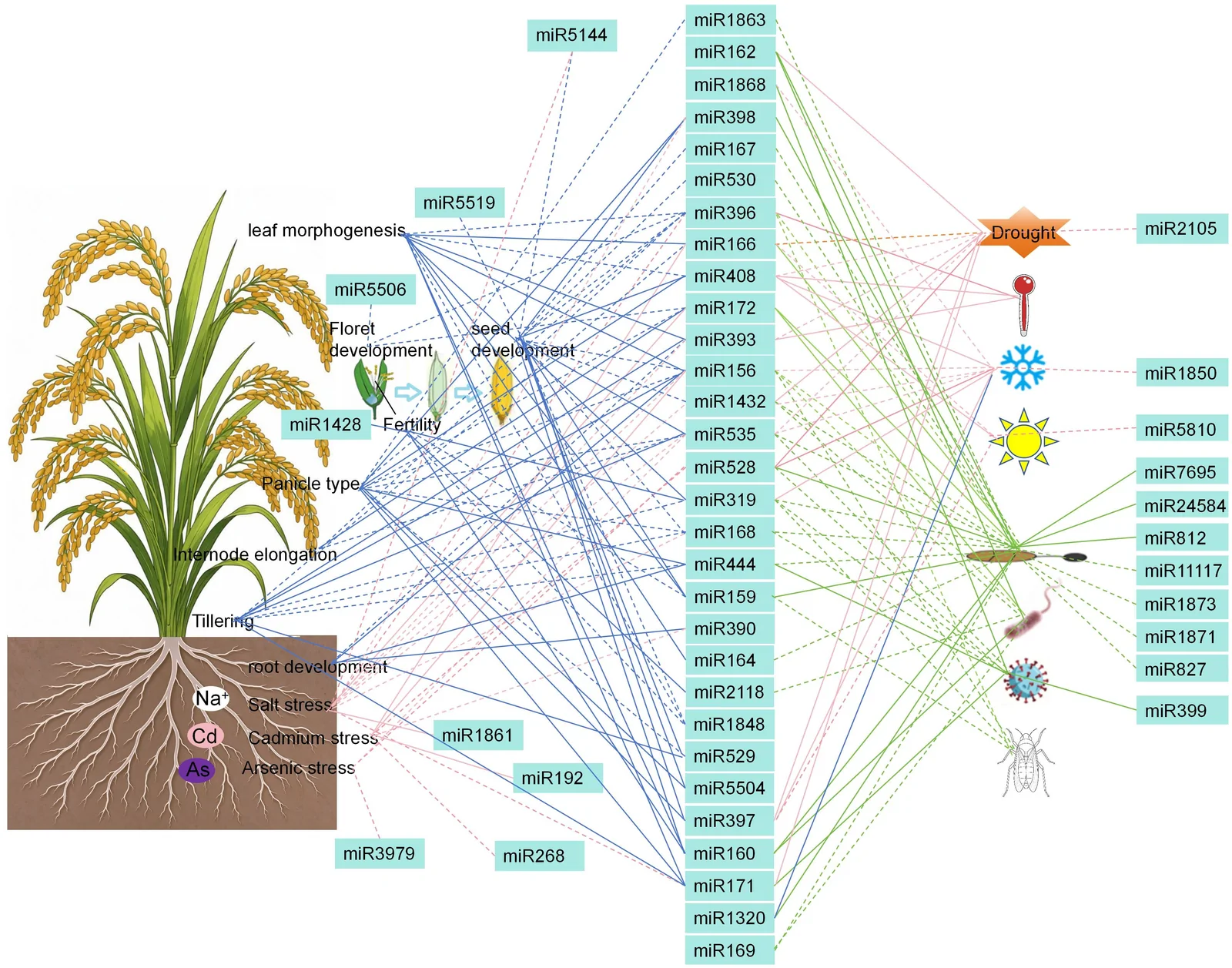
Summary of known miRNA-mediated regulation in rice growth, development, and stress tolerance. Solid lines: positive regulation; dashed lines: negative regulation. Blue, pink, and green lines denote regulation of growth and development, abiotic stress, and biotic stress, respectively.
